# Daily Moderate Exercise Is Beneficial and Social Stress Is Detrimental to Disease Pathology in Murine Lupus Nephritis

**DOI:** 10.3389/fphys.2017.00236

**Published:** 2017-04-26

**Authors:** Saba I. Aqel, Jeffrey M. Hampton, Michael Bruss, Kendra T. Jones, Giancarlo R. Valiente, Lai-Chu Wu, Matthew C. Young, William L. Willis, Stacy Ardoin, Sudha Agarwal, Brad Bolon, Nicole Powell, John Sheridan, Naomi Schlesinger, Wael N. Jarjour, Nicholas A. Young

**Affiliations:** ^1^Department of Internal Medicine Division of Rheumatology and Immunology, Ohio State University Wexner Medical CenterColumbus, OH, USA; ^2^Ohio State University Wexner Medical CenterColumbus, OH, USA; ^3^The Inflammation FoundationOrlando, FL, USA; ^4^The Biomechanics and Tissue Engineering Laboratory, College of Dentistry, Ohio State University Wexner Medical CenterColumbus, OH, USA; ^5^Department of Veterinary Biosciences and the Comparative Pathology and Mouse Phenotyping Shared ResourceColumbus, OH, USA; ^6^Institute for Behavioral Medicine Research, Ohio State University Wexner Medical CenterColumbus, OH, USA; ^7^Division of Rheumatology, Department of Medicine, Rutgers Robert Wood Johnson Medical SchoolNew Brunswick, NJ, USA

**Keywords:** lupus nephritis, psychological stress, cytokines, inflammation, exercise, histopathology

## Abstract

Daily moderate exercise (DME) and stress management are underemphasized in the care of patients with lupus nephritis (LN) due to a poor comprehensive understanding of their potential roles in controlling the inflammatory response. To investigate these effects on murine LN, disease progression was monitored with either DME or social disruption stress (SDR) induction in NZM2410/J mice, which spontaneously develop severe, early-onset LN. SDR of previously established social hierarchies was performed daily for 6 days and DME consisted of treadmill walking (8.5 m/min for 45 min/day). SDR significantly enhanced kidney disease when compared to age-matched, randomly selected control counterparts, as measured by histopathological analysis of H&E staining and immunohistochemistry for complement component 3 (C3) and IgG complex deposition. Conversely, while 88% of non-exercised mice displayed significant renal damage by 43 weeks of age, this was reduced to 45% with exercise. DME also reduced histopathology in kidney tissue and significantly decreased deposits of C3 and IgG complexes. Further examination of renal infiltrates revealed a macrophage-mediated inflammatory response that was significantly induced with SDR and suppressed with DME, which also correlated with expression of inflammatory mediators. Specifically, SDR induced IL-6, TNF-α, IL-1β, and MCP-1, while DME suppressed IL-6, TNF-α, IL-10, CXCL1, and anti-dsDNA autoantibodies. These data demonstrate that psychological stressors and DME have significant, but opposing effects on the chronic inflammation associated with LN; thus identifying and characterizing stress reduction and a daily regimen of physical activity as potential adjunct therapies to complement pharmacological intervention in the management of autoimmune disorders, including LN.

## Introduction

Lupus nephritis (LN), a debilitating clinical manifestation of systemic lupus erythematous (SLE) contributing to disease morbidity, affects more than 50% of adult and pediatric patients with lupus (Imran et al., 2015). The precise effects of psychological stress modification and exercise in autoimmune-mediated inflammatory disease, including LN, remain relatively unknown. However, many animal studies have shown that psychosocial stress induces a proinflammatory response (Powell et al., 2013b). In contrast, overwhelming experimental evidence from epidemiological studies has demonstrated that regular exercise correlates with an enhanced quality of life, increased life expectancy, and a reduced risk of mortality (Mokdad et al., 2004). Consequently, exercise and stress reduction are increasingly being emphasized as an adjunct to drug therapy because of their apparent disease modifying properties, but the mechanism(s) controlling disease pathobiology must be elucidated before becoming an accepted therapeutic strategy in the treatment of inflammatory diseases.

Our previous research has shown that psychological stressors and moderate exercise elicit opposing immunomodulatory effects. Murine restraint stress was shown to induce a proinflammatory response through enhanced levels of IL-6 (Voorhees et al., 2013). Moreover, social disruption stress (SDR) induced pulmonary inflammation via increased monocyte and neutrophil recruitment and activation (Curry et al., 2010). Conversely, we have also shown that daily moderate exercise (DME) can significantly reduce systemic inflammatory responses following lipopolysaccharide injection in mice by suppressing NF-κB activity (manuscript in review). Interestingly, these effects were transient and lost after 24 h, which suggests greater efficacy with a daily regimen. However, the influence of psychological stressors or daily moderate exercise on LN-associated inflammatory processes and disease pathology has yet to be comprehensively examined.

Since SLE pathogenesis involves high levels of autoimmune-mediated inflammation, this study aimed to investigate the effects of both moderate exercise and induced social stress on the inflammatory responses associated with LN in a murine model, NZM2410/J. These mice spontaneously develop severe, early onset glomerulonephritis from 20 to 40 weeks of age. Considering the age of NZM2410/J mice when they develop LN, we implemented the exercise regimen well before disease onset as a preventative measure and induced stress at or near the time of disease onset to potentially exacerbate disease progression. The objective was to test the hypothesis that SDR induces more severe inflammatory pathology while DME improves progression of kidney disease in order to provide supportive evidence for stress management and moderate exercise as adjunct therapeutic modalities for LN and other autoimmune diseases.

## Methods

### Animals

Female and male NZM2410/J and wild-type C57BL/6 control mice were obtained from The Jackson Laboratories (Bar Harbor, ME). Mouse maintenance and protocols were approved by the Institutional Animal Care and Use Committee at The Ohio State University Wexner Medical Center (OSUWMC). The animal facility was maintained at 22–23°C and 30–50% relative humidity with a 12-h light/dark cycle; chow and water were supplied *ad libitum*. blood urea nitrogen (BUN) levels were measured biweekly and weights were recorded weekly. As clinical indicators of kidney damage, experimental removal criteria was defined by a threshold of 20% weight loss and a BUN level above 50 mg/dL.

### Social disruption stress (SDR)

The SDR protocol was carried out as previously described (Avitsur et al., 2001). Briefly, an aggressor male mouse was introduced into a cage housing male NZM2410/J mice for 2 h daily for 6 days. This 6 day exposure results in well-established and characterized chronic stress response in mice (Powell et al., 2013a). If mice displayed visible signs of fighting or wounds following exposure to the aggressor, they were removed from the study. When a mouse undergoing the SDR protocol reached early removal criteria (BUN > 50 mg/dL; weight loss > 20%), an age-matched control NZM2410/J male mouse was randomly selected for comparative analysis. Experiments were performed separately and similar trends were observed in 2 experimental cohorts of *n* = 9 to demonstrate repeatability. Control NZM2410/J mice were housed in the same location and handled similarly under identical standard of care conditions, but not exposed aggressors.

### Daily moderate exercise (DME)

NZM2410/J female and male mice began exercise at the age of 11–13 weeks. Mice were exercised at 8.5 m/min, 45 min/day, and 7 days/week, with the exception of 1 day every 2 weeks for blood collection. Exercise was carried out using a multiple lane mouse treadmill (Columbus Instruments, Columbus, OH). Control mice were handled similarly, but not exercised on the treadmill. The primary endpoint of the exercise arm of the study was mortality; when mice displayed signs of terminal renal disease (BUN > 50 mg/dL; weight loss > 20%), kidney tissue and serum were collected. To demonstrate repeatability, experiments were performed twice in cohorts of *n* = 11 with similar trends observed.

### Bun level measurements

Serum was collected biweekly using the MaxDiscovery Blood Urea Nitrogen Enzymatic Assay Kit (Bioo Scientific Corporation, Austin, TX) according to manufacturer's protocol. Blood was obtained via submandibular bleeds and serum was isolated by centrifugation after blood clotting. Absorbance values were determined using the Dynex MRX-TC Revelation microplate reader/colorimeter (Dynex Technologies, Chantilly, VA). Results were exported to Microsoft excel (v2013) for analysis.

### ELISA (enzyme-linked immunosorbent assay)

Blood was obtained via submandibular bleeding or axillary vessel incision at the time of sacrifice. Cytokine analysis on collected serum was done using electrochemiluminescence detection (V-PLEX Proinflammatory Panel 1 mouse kit; Meso Scale Diagnostics, Rockville, MD) per manufacturer's protocol. Anti-dsDNA ELISAs were performed following manufacturer's instruction using the Mouse Anti-dsDNA kit (Signosis, Sunnyvale, CA). Serum from SDR experimental endpoint was compared with a randomly selected, age-matched control (non-stressed) NZM2410/J mouse. For analysis of DME, relative changes in expression were analyzed over a 2 month period of bi-weekly measurements. MCP-1 ELISAs were performed using Mouse CCL2 (MCP-1) ELISA Ready-SET-Go! (eBioscience Inc., San Diego, CA) according to manufacturer's protocol.

### Histopathology and image analysis

Mouse tissues were dissected for paraffin processing according to previously established protocol (Young et al., 2014). Serial paraffin sections were used for immunohistochemistry and hematoxylin and eosin (H&E) staining was performed as detailed formerly (Young et al., 2013). Briefly, slides were stained in Richard Allan Scientific Hematoxylin (Thermo Scientific, Waltham, MA) and Eosin-Y (Thermo Scientific) with the Leica Autostainer (Leica Biosystems, Buffalo Grove, IL). Immunohistochemical (IHC) staining was performed using rat anti-mouse F4/80 (1:200; AbD Serotec, Raleigh, NC), rat anti-mouse C3 (1:50; Abcam, San Francisco, CA), or goat anti-mouse IgG (1:60,000; Jackson Immunoresearch, West Grove, PA) polyclonal primary antibodies for 1 h at room temperature with the Intellipath Autostainer Immunostaining instrument. Horseradish peroxidase (HRP)-conjugated secondary antibodies included the following: rabbit anti-Goat (1:200; Abcam) in 2% Normal Goat serum (Vector Labs, Burlingame, CA) and goat anti-rat (1:200, Abcam).

Inflammation severity and histopathological scoring was performed blindly by a board-certified veterinary pathologist (BB) using the 10x objective of a bright-field light microscope. Scoring criteria were defined as: 0 = within normal limits (glomeruli: modest numbers of elongated oval cells within loops, minimal amount of mesangial matrix, no leukocytes; interstitium: essentially no mononuclear cells near glomeruli or tubules; tubules: segments are lined by uniform cuboidal epithelium with eosinophilic cytoplasm, does not contain eosinophilic, protein-rich casts); 1 = minimal nephritis (glomeruli: some to many contain a few mononuclear leukocytes and/or slightly expanded mesangial matrix; interstitium: a few mononuclear cells are located near affected glomeruli or tubules; tubules: a few segments are lined by attenuated flat cuboidal epithelium with basophilic cytoplasm and/or contain casts); 2 = mild nephritis (glomeruli: many contain a diffuse infiltrate of mononuclear leukocytes without aggregates and/or substantially expanded mesangial matrix; interstitium: a few mononuclear cells, including small clusters, are located near affected glomeruli or tubules; tubules: clusters of segments, especially in the outer cortex, are lined by attenuated epithelium); 3 = moderate nephritis (glomeruli: most contain a diffuse infiltrate of mononuclear leukocytes including small aggregates and/or substantially expanded mesangial matrix; interstitium: many mononuclear cells, including small to large clusters, are located near affected glomeruli or tubules; tubules: large clusters of segments, especially in the outer cortex, are lined by attenuated epithelium, and about 5% of tubules contain casts); and 4 = marked nephritis (glomeruli: most are visibly distorted by a diffuse infiltrate of mononuclear leukocytes, including large aggregates, and/or substantially expanded mesangial matrix; interstitium: many mononuclear cells, with many large clusters, are located near affected glomeruli or tubules; tubules: large clusters of segments, especially in the outer cortex, are lined by attenuated cuboidal epithelium, and many tubules contain casts).

Slides were digitally scanned using the Aperio ScanScope XT eSlide capture device (Aperio, Vista, CA) and analyzed using Aperio Digital Image Analysis software (v9.1) to measure pixel intensities according to previously described methods (Young et al., 2014). For quantitation of IgG and complement component 3 (C3) protein, the intensity of staining in the glomeruli was specifically assessed after subtraction of non-specific binding. For F 4/80, total renal staining (tubulointerstitium and glomeruli) was measured after normalization accounting for nonspecific binding.

### Statistics

All numerical data were expressed as mean values with standard error of the mean (SEM) using Graph Pad Prism 6. Statistical analysis for two groups was performed using unpaired, two-tailed nonparametric Mann-Whitney U test or unpaired, two-tailed parametric Student's *t*-tests where indicated. Statistical analysis for three groups was performed using one-way ANOVA followed by Tukey's *post hoc* test. Data was considered statistically significant if *p* ≤ 0.05.

## Results

### Repeated social stress significantly enhances lupus nephritis pathology

To examine the effects of psychosocial stressors on chronic autoimmune-mediated inflammation, NZM2410/J mice were subjected to the SDR model as previously described (Avitsur et al., 2001) at 19–23 weeks of age; BUN levels and weights were monitored. This model has been shown to produce a chronic stress response in mice, which has been previously characterized and extensively published on by our research team for more than 15 years (Powell et al., 2013a). To assess renal disease, early removal criteria was defined by a threshold of 20% weight loss and a BUN level above 50 mg/dL. When a mouse undergoing the SDR protocol met the early removal criteria, kidney tissue and serum were collected for comparison with a random age-matched control.

**Figure 1:**
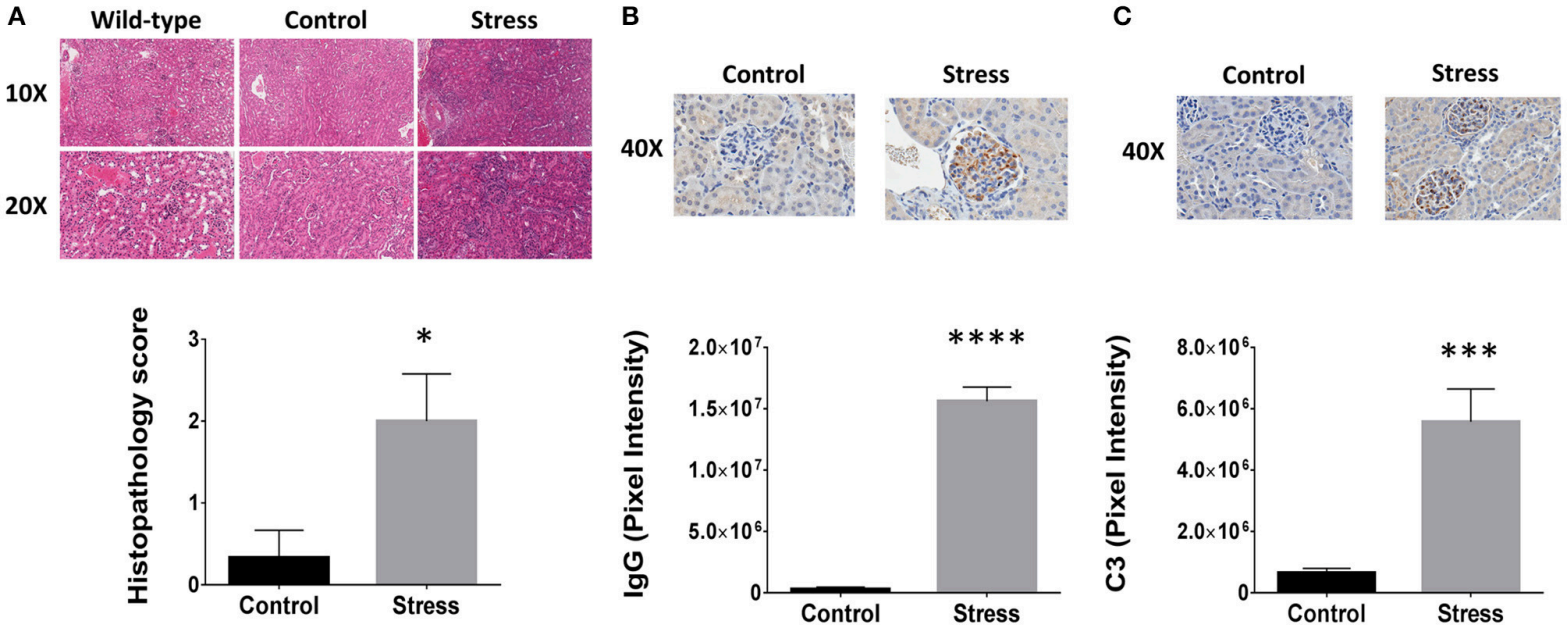
**Psychological stressors enhance lupus nephritis pathology in NZM2410/J mice**. To disturb the order of an already established social hierarchy, an aggressor was introduced to induce repeated social disruption stress (SDR). Each time a mouse undergoing SDR (*n* = 9) met the experimental removal criteria (BUN > 50 mg/dL; weight loss > 20%), an age-matched control counterpart was selected at random for comparative analysis. **(A)** H&E staining (top) of stressed and non-stressed NZM2410/J mice for comparison to wild-type C57BL/6 controls (*n* = 3). Histopathological scoring analyzed by Mann-Whitney *U*-test. (bottom). **(B,C)** Immunohistochemical staining (top) and Aperio digital image analysis by *T*-test (bottom). **(B)** IgG or **(C)** complement component 3 (C3) protein. Results representative of trends observed in duplicate experiments. Values are the mean ± SEM. ^*^p < 0.05; ^***^p < 0.001; ^****^p < 0.0001 vs. non-stressed controls.

H&E staining revealed that SDR induced infiltration of mononuclear leukocytes when compared to both control and wild-type mice (Figure 1A). The inflammatory pathology is characterized by glomerular hypercellularity and hyperplasia of the Bowman's capsule. Confirmation of kidney disease was made by a blinded analysis performed by a board certified veterinary pathologist and concluded that SDR induced significantly greater kidney pathology relative to control mice, which remained within normal levels (Figure 1A). While the control mice had an average score of 0.33 ± 0.5, those in the SDR group scored 2.0 ± 1.0 (*p* < 0.038). Additional IHC staining was done to further investigate the inflammatory components found in the renal tissues. Specifically, immune complexes and C3 protein were measured because of the strong association with renal injury in LN; inflammatory pathology of LN is characterized by activation of the compliment cascade, which leads to deposition of C3 fragments on target cells. IHC analysis showed that SDR resulted in over 50-fold more IgG complex deposition (*p* < 2 × 10^−7^; Figure 1B) and over 8-fold higher C3 staining (*p* < 0.001; Figure 1C).

### Daily moderate exercise significantly reduces inflammatory disease in mice with lupus nephritis

NZM2410/J mice were exercised daily at moderate intensity by treadmill walking beginning at 11–13 weeks of age (8.5 m/min for 45 min) to determine the influence of exercise on LN disease progression. Control mice were handled similarly, but not exercised on the treadmill. When mice displayed signs of terminal renal disease (BUN > 50 mg/dL; weight loss > 20%), kidney tissue and serum were collected for analysis.

**Figure 2:**
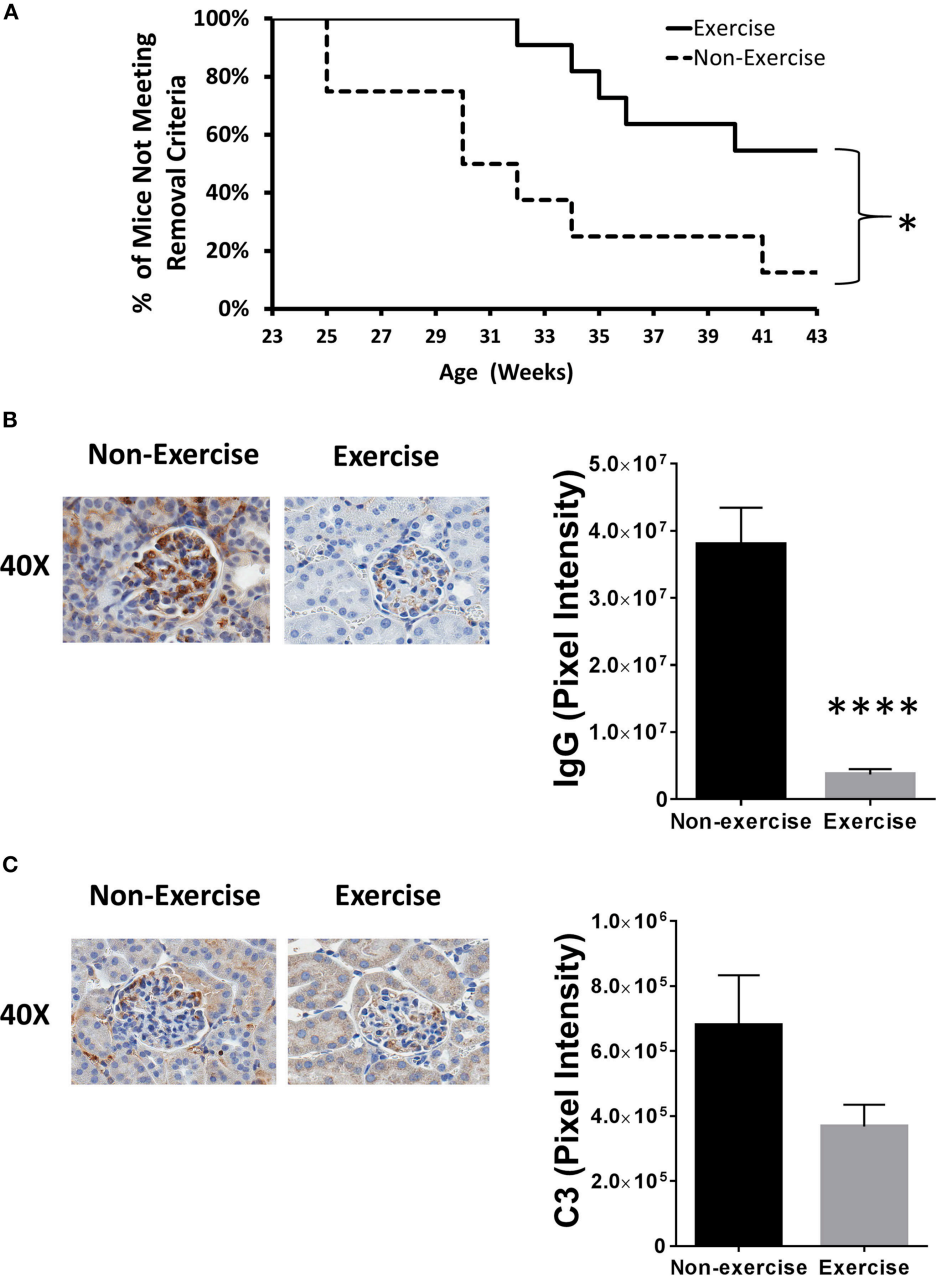
**Exercise mitigates lupus nephritis disease progression in the NZM2410/J animal model**. Mice (*n* = 11) were exercised daily for 45 min by treadmill walking at moderate intensity (8.5 m/min) beginning at 11–13 weeks of age and compared to non-exercised controls (*n* = 8). **(A)** Kaplan–Meier plot showing when mice were removed from the study due to meeting early removal criteria (BUN > 50 mg/dL; weight loss > 20%). **(B,C)** Immunohistochemical staining (left) and Aperio digital image quantitation (right). **(B)** IgG or **(C)** complement component 3 (C3) protein. Experiments repeated in duplicate. Values are the mean ± SEM. Significant differences derived from a nonparametric Mann-Whitney *U*-test. ^*^p < 0.05; ^****^p < 0.0001 vs. non-exercised controls.

After 43 weeks of age, DME reduced severe kidney damage incidence by 38% and mice meeting experimental removal criteria differed significantly throughout the course of experimentation when plotted on a Kaplan–Meier curve (*p* < 0.05; Figure 2A). Relative to baseline levels taken prior to both disease onset and DME, BUN levels were 4.6-fold higher (*p* < 0.05) in the non-exercised (11 mg/dL ± 4.3) mice at 32 weeks of age when compared to mice that were exercised (2.4 mg/dL ± 1.2). To further confirm changes in inflammatory pathology, IHC for C3 protein and antibody complexes of IgG were quantified. Exercised mice showed over 10-fold reduction in positive staining for IgG antibodies (*p* < 1 × 10^−4^; Figure 2B). Decreased C3 deposition was also detected in exercised mice (2-fold), but the difference was not statistically significant (Figure 2C).

### Social stress and moderate exercise both influence inflammatory mediator expression in NZM2410/J mice

**Figure 3:**
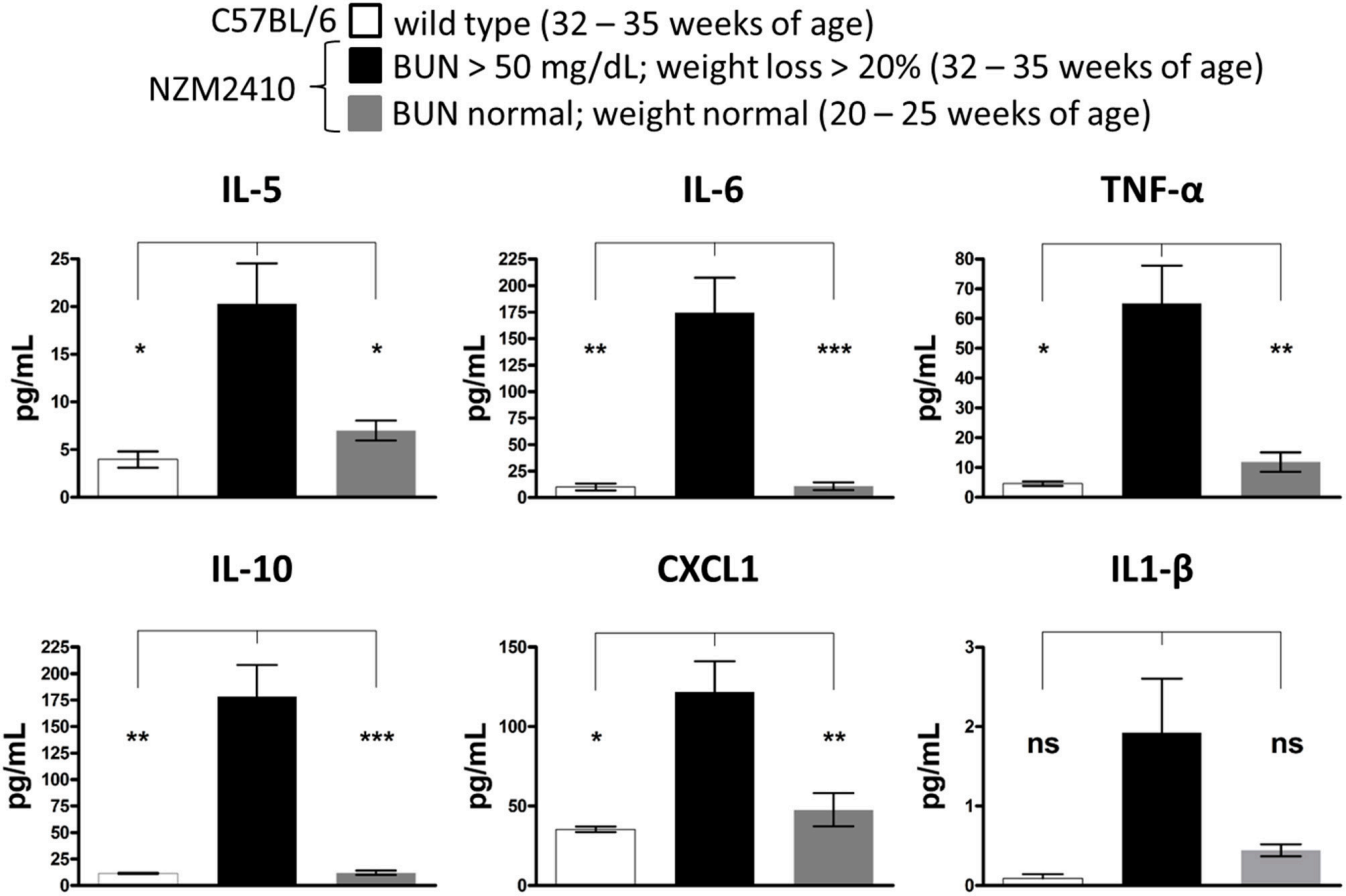
**Expression of proinflammatory cytokines is associated with disease progression in NZM2410/J mice**. As clinical indicators of kidney disease progression, weights were recorded weekly and blood urea nitrogen (BUN) levels were measured bi-weekly in NZM2410/J mice (*n* = 8 per experimental condition). When mice met experimental removal criteria (BUN > 50 mg/dL; weight loss > 20%), serum was collected for comparative analysis with both younger mice not meeting these criteria and wild-type C57BL/6 controls (*n* = 3). Expression was measured using electrochemiluminescence ELISA for the cytokines indicated. Values are the mean ± SEM. Significant differences derived from a one-way ANOVA followed by Tukey's *post hoc* test. ^*^p < 0.05; ^**^p < 0.01; ^***^p < 0.001 vs. NZM2410/J mice meeting removal criteria, ns = not significant.

To determine an association between serum cytokine expression and LN disease progression in the NZM2410/J mouse model, serum was collected from mice meeting early removal criteria (BUN > 50 mg/dL; weight loss > 20%) for comparison to age-matched wild-type controls (32–35 weeks) and younger NZM2410/J mice (20–25 weeks) not yet meeting these criteria. While serum levels of IFN-γ, IL-2, IL-4, and IL-12 did not display a difference between experimental groups (data not shown), expression of IL-5, IL-6, TNF-α, IL-10, CXCL1, and IL-1β was elevated in the NZM2410/J mice displaying signs of severe kidney damage (Figure 3). Specifically, upregulation was observed in the levels of IL-5 (5-fold; *p* < 0.05), IL-6 (17-fold; *p* < 0.01), TNF-α (14-fold; *p* < 0.05), IL-10 (15-fold; *p* < 0.01), and CXCL1 (3.4-fold; *p* < 0.05) relative to wild-type mice (Figure 3). When compared to NZM2410/J mice that did not meet experimental removal criteria, mice showing signs of kidney dysfunction had significantly higher IL-5 (2.8-fold; *p* < 0.05), IL-6 (16-fold; *p* < 0.001), TNF-α (6-fold; *p* < 0.01), IL-10 (15-fold; *p* < 0.001), and CXCL1 (2.5-fold; *p* < 0.01) (Figure 3).

**Figure 4:**
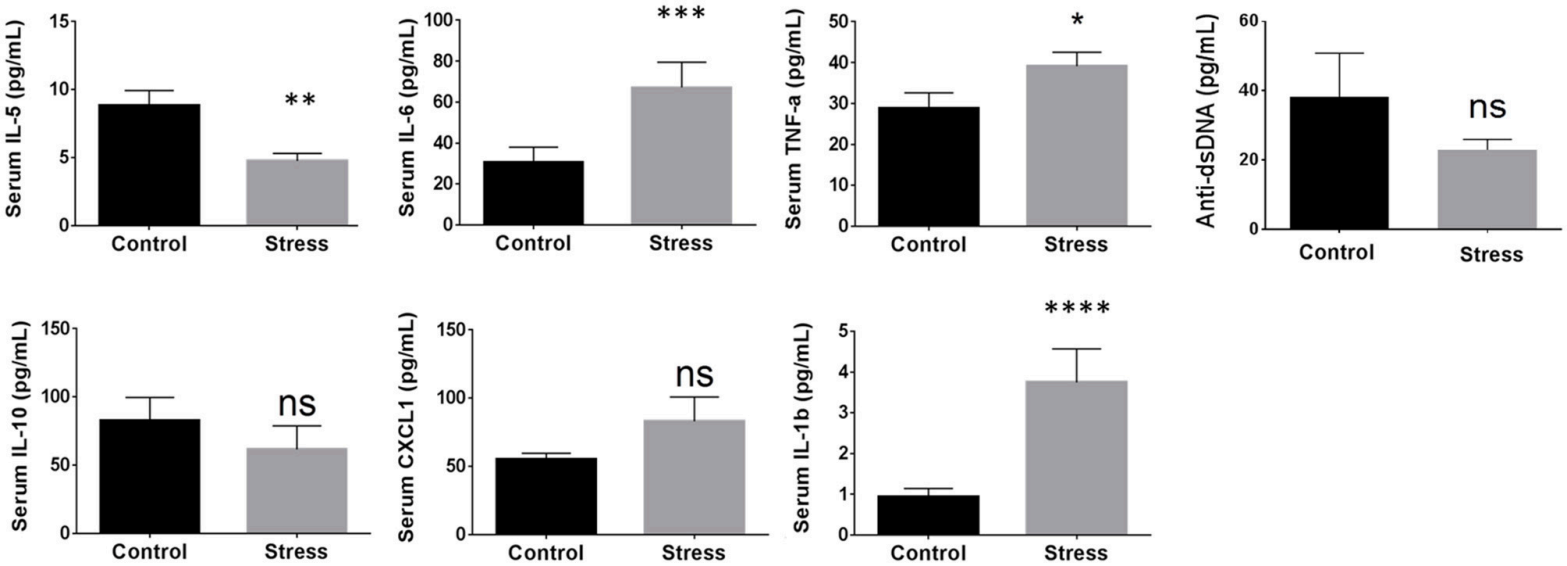
**Stress induced by social disruption (SDR) influences circulating proinflammatory mediator secretion in NZM2410/J mice**. SDR was carried out according to the protocol outlined in the methods section (*n* = 9 per experimental condition) on mice 19–23 weeks of age. When a mouse in the SDR cohort reached experimental removal criteria (BUN > 50 mg/dL; weight loss > 20%), an age-matched control was selected at random that was not exposed to SDR. Serum was isolated from whole blood for analysis of the indicated cytokines or for anti-dsDNA autoantibody levels by ELISA. Values are the mean ± SEM. Significant differences derived from a nonparametric Mann-Whitney *U*-test. ^*^p < 0.05; ^**^p < 0.01; ^***^p < 0.001; ^****^p < 0.0001 vs. non-stressed controls.

**Figure 5:**
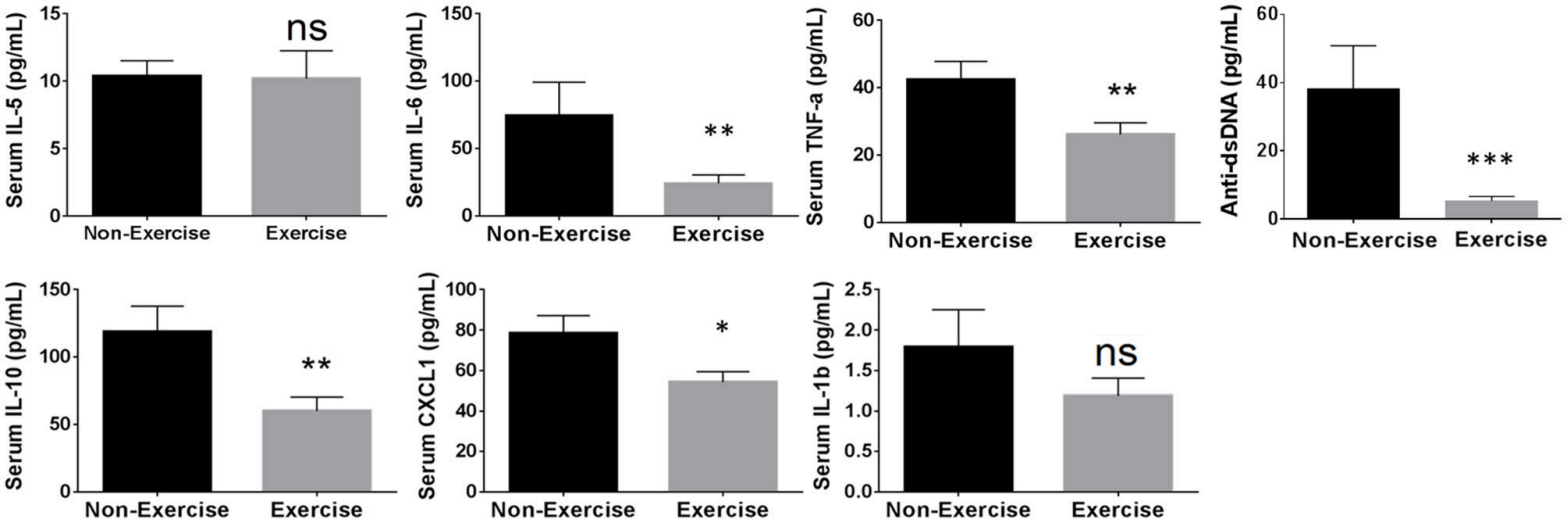
**Daily moderate exercise (DME) of NZM2410/J mice suppresses expression of proinflammatory mediators**. Beginning at 11–13 weeks of age, mice were exposed to DME consisting of treadmill walking (8.5 m/min, 45 min/day) for comparative analysis with non-exercised control mice. Serum was collected bi-weekly for longitudinal analysis and weights were recorded weekly (*n* = 9 per experimental condition). ELISA measurements from serum were used to determine average cytokine scores from samples collected at experimental endpoint (BUN > 50 mg/dL; weight loss > 20%) and the 3 previous time points for each mouse. Values are the mean ± SEM. Significant differences derived from a nonparametric Mann-Whitney *U*-test. ^*^p < 0.05; ^**^p < 0.01; ^***^p < 0.001 vs. non-exercised controls.

Using the cytokines identified above showing correlation to disease progression, the influence of SDR and DME was examined. In addition to cytokines, anti-dsDNA autoantibody levels were measured because this has been shown previously to correlate with glomerulonephritis in NZM2410/J mice (Mohan et al., 1998). SDR resulted in a significant enhancement of cytokine expression, including IL-6 by 2.19-fold (*p* < 0.0003; *U* = 247), TNF-α by 1.4-fold (*p* < 0.0244; *U* = 356), and IL-1β by 3.4-fold (*p* < 0.0001; *U* = 63); IL-10, CXCL1, and anti-dsDNA autoantibody levels did not change significantly and IL-5 expression was downregulated (Figure 4). Since the onset and progression of renal disease varies from mouse to mouse in this model, inflammatory mediators were measured over 2 months to display cumulative expression over a broader period of time in the longitudinal analysis of DME. Thus, these average scores more effectively express the overall extent of activation and expression of each proinflammatory cytokine over a larger window of time. DME resulted in a significant decrease in IL-10 by 1.94-fold (*p* < 0.0082; *U* = 441), IL-6 by 3.1-fold (*p* < 0.0046; *U* = 424), CXCL1 by 1.45-fold (*p* < 0.0150; *U* = 460), TNF-α by 1.62-fold (*p* < 0.0062; *U* = 433), and anti-dsDNA autoantibodies by 7.2-fold (*p* < 0.0007; *U* = 255), while no significant changes were observed with IL-5 or IL-1β production (Figure 5).

### Modulation of macrophage-mediated kidney inflammation by social stress induction or moderate exercise

**Figure 6:**
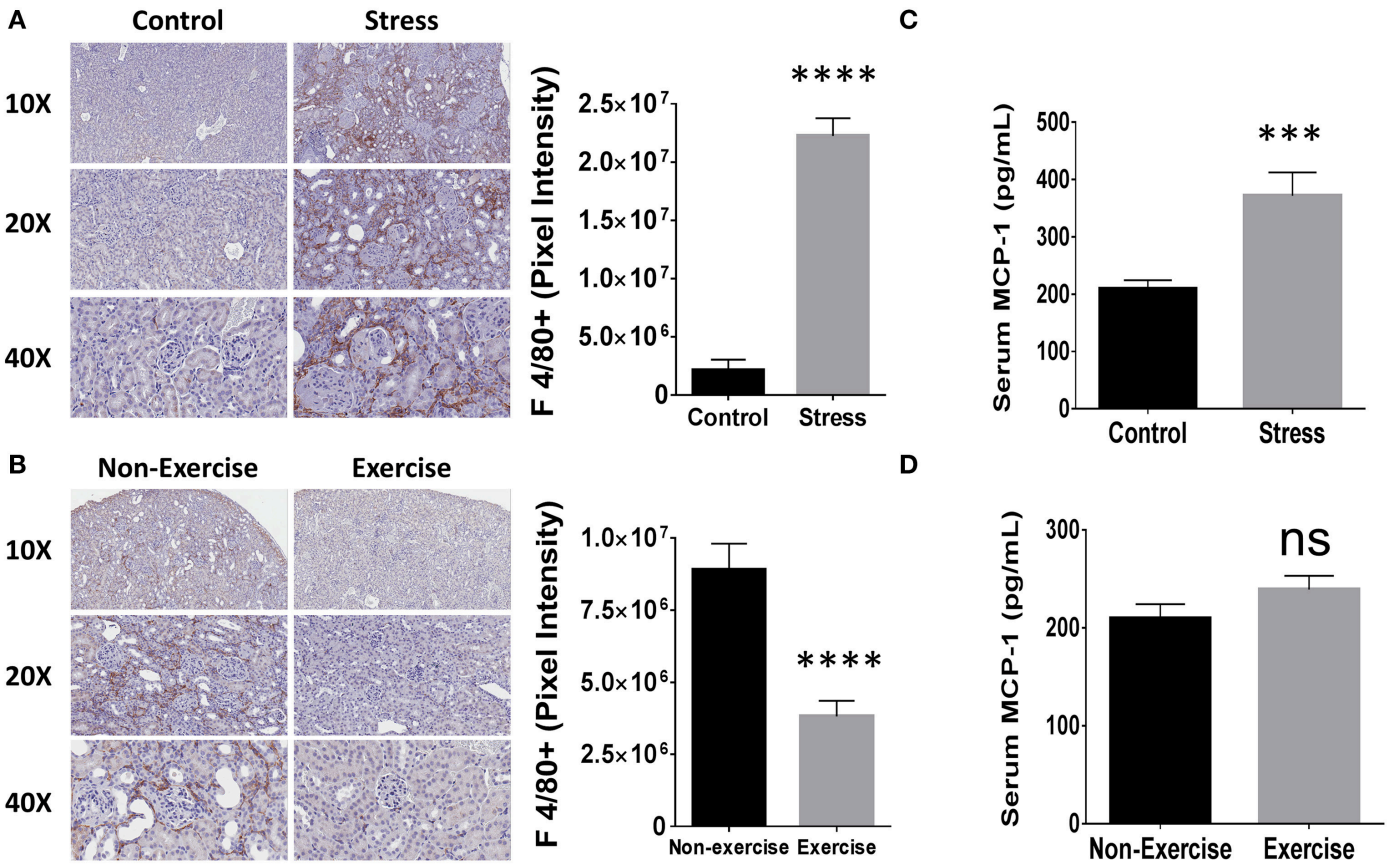
**Social stress (SDR) and moderate exercise (DME) are associated with altered macrophage-mediated renal inflammation and levels of MCP-1. (A,B)** Kidney tissue (*n* = 3 per experimental condition) was collected at experimental endpoint and fixed for paraffin-embedding and immunhistochemical staining for macrophage marker F 4/80. Representative images from Aperio slide scanning (left) and digital quantitation of pixel intensity corresponding to positive staining (right). **(A)** SDR or **(B)** DME. **(C,D)** MCP-1 expression measured by ELISA (*n* = 8). **(C)** SDR or **(D)** DME. Values are the mean ± SEM. ^***^p < 0.001; ^****^p < 0.0001 vs. control condition by *T*-test, ns = not significant.

Since mononuclear cell infiltration in the kidney correlates with poor disease outcome in human LN patients (Hill et al., 2001) and macrophage activation is associated with disease progression in NZM2410/J mice (Bethunaickan et al., 2014), the influence of SDR and DME on macrophage-mediated kidney inflammation was investigated. To determine the influence of repeated social stress and daily moderate exercise on macrophage infiltration, IHC was performed on kidney tissue sections for a common marker of mouse macrophages, F 4/80. Mice from the SDR cohorts showed a 10.5-fold increase in staining for this marker compared to control counterparts (*p* < 3 × 10^−5^) and macrophages were detected primarily in the interstitial regions (Figure 6A). Furthermore, DME reduced macrophage presence in the tubulointerstitium by 3-fold relative to non-exercised controls (*p* < 4 × 10^−7^; Figure 6B).

Monocyte chemoattractant protein (MCP)-1 promotes macrophage-mediated inflammation and its association to renal pathology in autoimmunity is specifically evidenced by elevated levels in the urine of patients with LN (Noris et al., 1995). Furthermore, MCP-1 is upregulated similarly both in the serum and kidney tissue of NZM2410/J mice as autoimmune-mediated glomerulopathy progresses (Suzuki et al., 2012). Serum levels were measured in the SDR and DME experimental cohorts to determine the effects on systemic expression of MCP-1. Relative to randomly selected controls of the same age, SDR induced the upregulation of MCP-1 expression by 1.9-fold (*p* < 5 × 10^−4^; Figure 6C). Levels of MCP-1 did not differ significantly between exercised or non-exercised mice and were similar to SDR controls (Figure 6D).

## Discussion

Physicians recommend that patients with SLE engage in 30–50 min of exercise approximately 3 times a week due to the association of exercise with improved patient outcome in various studies (Yuen and Cunningham, 2014), but optimal frequency, duration, and extent have yet to be sufficiently characterized. Furthermore, stress reduction programs offered to patients with SLE have demonstrated improved disease activity (Williams et al., 2014) and literature shows the positive effects of exercise in improvement of SLE disease management and clinical outcome (Ayán and Martín, 2007). However, the counseling regarding regular exercise and stress modification programs varies among physicians and there is no evidence-based best practice currently. Our data characterize the effects of SDR and DME on murine LN in the NZM2410/J model and demonstrate that SDR significantly exacerbates the inflammatory process whereas this response is dramatically reduced with DME. This is the first study to our knowledge to characterize the histopathological and molecular effects of both exercise and psychosocial stressors on the autoimmune-mediated inflammation of lupus nephritis; our results suggest that stress reduction and DME may be beneficial supplemental therapies to be applied in adjunct to current pharmacologic treatments of LN.

Chronic inflammation is a contributing factor to localized and systemic disease pathogenesis and is influenced by both psychosocial stressors and physical exercise. Therefore, characterizing their immunomodulatory effects could have far-reaching clinical implication. Our results show that expression of TNF-α, IL-6, and IL-10 significantly increases with the development of more severe lupus-like illness in NZM2410/J mice, which is in agreement with studies examining cytokine expression in human SLE (Chun et al., 2007). In addition to significant deposition of IgG and C3 complexes, the NZM2410/J mice exposed to SDR express chronically high levels of TNF-α, IL-6, and IL-1β. In contrast, DME resulted in significant reductions in renal deposition of IgG and C3 as well as circulating levels of TNF-α, IL-6, IL-10, and CXCL1. Although exercise is recognized for combating the stress and fatigue commonly reported by patients with SLE (Ayán and Martín, 2007), IL-6 is shown to increase immediately following acute bouts of exercise, presumably via secretion from contracting skeletal muscle as a myokine (Perandini et al., 2015). Our data displays a prolonged suppression of IL-6; thus, while the acute response to exercise may lead to an increase, the chronic influence is to suppress this proinflammatory mediator. Furthermore, we observed a significant increase in IL-6 levels with SDR in NZM2410/J mice compared to non-stressed controls. In concordance, high levels of circulating IL-6 are strongly associated with stress, depression, and autoimmune disease (Rohleder et al., 2012). IL-10 has been shown previously to be elevated in SLE and is known to have an anti-inflammatory function by acting to inhibit macrophage functions and T-cell proliferation; however, it is also known to be proinflammatory by recruiting cytotoxic T cells (Santin et al., 2000). While we did not observe a difference in IL-10 following SDR, significant reductions were observed with DME. Since IL-10 upregulation is also associated with disease progression in NZM2410/J mice (Blenman et al., 2006), our data suggests that the effects of SDR may be masked by prevailingly elevated IL-10, whereas DME is effective in reducing these levels. Collectively, these data suggest that DME and stress reduction may be effective in combating the inflammatory disease pathology associated with these cytokines.

Previous studies have shown correlations between mononuclear cell infiltration and poor disease outcome in patients with LN (Hill et al., 2001). In addition to high levels of antinuclear antibodies and immune complex formation, levels of MCP-1 are also observed to increase with SLE flare (Bauer et al., 2009). Furthermore, TNF-α is known to act as a promotor for MCP-1 expression in humans as well as mice (Ping et al., 1996; Hill et al., 2001; Bethunaickan et al., 2014), which leads to macrophage migration and infiltration (Deshmane et al., 2009). Since macrophage activation is being increasingly associated with disease progression in NZM2410/J mice (Bethunaickan et al., 2014) and our results show a significant immunomodulatory influence of TNF-α and macrophage infiltration with both DME and SDR, it is tempting to speculate that regular exercise and stress modification impact LN pathology via TNF-α regulation. Furthermore, our results suggest that the TNF-α upregulation resulting from SDR may lead to the induction of mononuclear cell infiltration by inducing MCP-1 expression. However, pharmacological suppression of TNF-α is not used regularly in patients with SLE (Aringer et al., 2012) despite several small studies demonstrating a significant improvement in disease activity scores (SLEDAI) with anti-TNF-α therapy (Zhu et al., 2010). While the reasoning to explain why no large randomized trials have been conducted to examine these observations is complex, one factor is the concern over rising anti-dsDNA autoantibody levels in some patients (Zhu et al., 2010). Considering that we observed a significant influence of TNF-α with SDR and DME without an increase in anti-dsDNA autoantibody levels, DME and psychological stress management may be natural and more efficacious as adjunct therapeutic strategies than pharmacologically inhibiting TNF-α.

The process of inflammation requires substantial energy expenditure, which has been demonstrated in animal studies using transgenic mice targeting NF-κB, a key transcriptional regulator of inflammatory signaling pathways (Tang et al., 2010). Furthermore, recent work in humans has demonstrated that physiological energy allocation is not additive with increasing physical activity, but rather follows a constrained energy expenditure model of distribution (Pontzer et al., 2016). According to this hypothesis, if physical activity is introduced or the system has other new energy demands, energy is re-allocated from different areas in order to satisfy these additional burdens. Considering the immunosuppressive influences observed by DME in this study, the compensatory changes required to maintain energy homeostasis may significantly impact the energy allocation provided to fuel systemic inflammation. In agreement, studies have shown that regular moderate exercise leads to decreased susceptibility to infectious disease (Nieman, 1997; Chubak et al., 2006). Therefore, by fine-tuning energy expenditure through regular physical activity, it may be possible to optimize immune system function. This immuno-optimization may both permit sufficient eradication of pathogenic challenges without producing clinical symptoms and limit the inflammatory pathology produced by autoimmune-mediated processes. Additionally, while food intake was not monitored in this study, the effect that this could potentially have on energy allocation is minimal considering the moderate intensity of physical activity and *ad libitum* availability of chow for mice.

The results of our study indicate that an ideal regimen to determine the efficacy of moderate exercise and stress management in improving disease outcomes through the regulation/suppression of systemic inflammation should incorporate both daily, moderate physical activity as well as techniques to promote psychological stress management. Accordingly, Tai Chi uses meditative exercise that incorporates both moderate physical activities as well as stress reduction; thus, may effectively translate in a study with lupus patients. In a small pilot study “Stress Moderation Impacting Lupus with Exercise (SMILE): influence of daily moderate exercise and stress reduction by Tai Chi in lupus,” active SLE patients were enrolled into a daily Tai Chi program, which emphasized moderate exercise levels with meditative breathing to provide daily physical activity and stress reduction (unpublished observations). Our preliminary data indicate that daily Tai Chi can potentially be an effective adjunct therapy to compliment current pharmacological interventions. Future investigations will expand SMILE study enrollment for a large randomized trial and further examine the molecular pathways regulated by SDR and DME in mice. Studies to better characterize these pathways may be especially advantageous in patients where behavioral modifications to alter psychological stress and physical activity levels may not be feasible.

## Ethics statement

Animal subjects: This study was carried out in accordance with the recommendations of the University Laboratory Animal Resources at The Ohio State University Wexner Medical Center. The protocol was approved by the Institutional Animal Care and Use Committee.

## Author contributions

Conceived and designed the experiments of the study: SA, JH, MB, SAg, NP, JS, WJ, and NY; Data collection and analysis: SA, MB, JH, BB, and NY; Performed experiments: SA, MB, JH, KJ, and NY; Edited manuscript: SA, JH, MB, KJ, GV, LW, MY, WW, SAr, SAg, BB, NP, JS, NS, WJ, and NY; Statistical assessments: SA, BB, and NY; Wrote manuscript: SA and NY; Contributed reagents/materials/analysis tools: BB, SAg, and WJ; Made substantial, direct and intellectual contribution to the work, and approved it for publication: SA, JH, MB, KJ, GV, LW, MY, WW, SAr, SAg, BB, NP, JS, NS, WJ, and NY.

## Funding

The CCTS was supported by Award Number Grant UL1TR001070 from the National Center for Advancing Translational Science. The CPMP was supported by grant P30 CA016058 from the National Cancer Institute. This study was also supported directly by OSUWMC funds. Additionally, NY and NS have research support from Ironwood Pharmaceuticals examining the role of exercise in inflammation and The Ohio State University is supported in part by a grant from the Lupus Research Alliance, which indirectly supported these research endeavors. The authors declare that they have funding from Ironwood Pharmaceuticals to explore the immunomodulatory effects of exercise, which indirectly contributed to this research. The funder was not involved in the study design or collection, analysis, or interpretation of the data.

### Conflict of interest statement

The authors declare that the research was conducted in the absence of any commercial or financial relationships that could be construed as a potential conflict of interest. The reviewer AB and handling Editor declared their shared affiliation, and the handling Editor states that the process nevertheless met the standards of a fair and objective review.
